# Effect of Sn Purity and Electropolishing Procedure on the Morphology and Properties of Nanoporous SnO_*x*_ Layers Obtained via Galvanostatic Anodization

**DOI:** 10.1021/acsomega.4c03630

**Published:** 2025-01-14

**Authors:** Magdalena Gurgul, Bernadetta Macuda, Tomasz Kuciel, Leszek Zaraska

**Affiliations:** Faculty of Chemistry, Jagiellonian University, Gronostajowa 2, Krakow 30-387, Poland

## Abstract

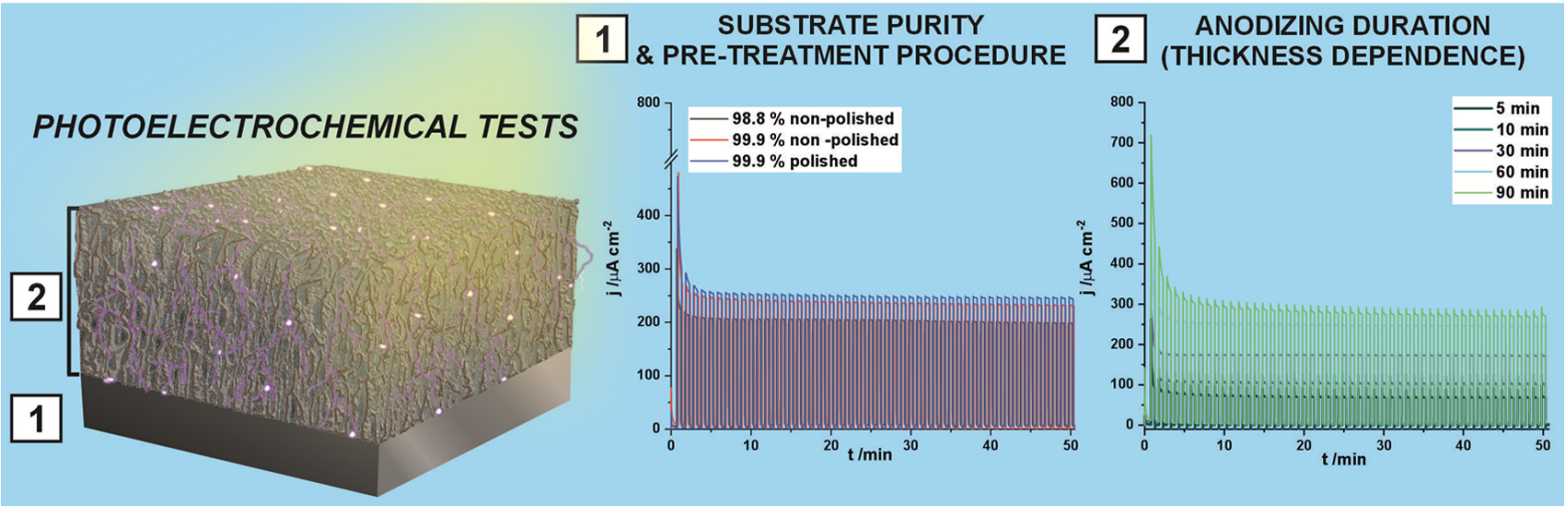

In this study, for the first time, we investigated the influence of Sn foil purity and electropolishing pretreatment procedure on the growth of nanoporous SnO_*x*_ fabricated during galvanostatic anodization. For this reason, anodic tin oxide layers were fabricated on different Sn foil substrates in a time range from 5 to 90 min, and a detailed inspection of morphological features of as-grown films was carried out using scanning electron microscope (SEM) and Atomic force microscope (AFM) microscopy. Afterward, a comparative investigation of optical and photoelectrochemical properties depending on the time of anodization and the substrate type was elucidated and discussed. The findings of the study indicate that the type of substrate influences the morphology and properties of the material, with the effect becoming more pronounced as the thickness of SnO_*x*_ increases.

## Introduction

Over the past decades, anodic oxidation of metals has gathered keen interest as an effective and economically benign path to fabricate various types of nanostructured metal oxides, possessing different dimensions and morphologies including nanowires,^1^ nanotubes,^2^ nanosheets,^3^ and many others. Among numerous materials that can be successfully obtained via this technique, nanoporous tin oxide is a semiconductor of particular significance that warrants further examination and exploration due to the wide range of its possible applications, e.g., in solar cells,^4^ Li-ion/Na-ion batteries,^5^ and gas sensors.^6^ With its promising performance, anodic SnO_*x*_ has also become an attractive candidate as a potential photoactive material for photoelectrochemical water-splitting devices.^7^

Current studies on anodic tin oxide revealed that it is possible to tailor some of the structural features and architecture (and so some of its properties) by simply adjusting anodizing conditions (mainly type of electrolyte, duration of the process, applied potential, or current) or by implementing additional post-treatment strategies (thermal treatment or soaking in water).^8^ Hence, it is crucial to deliberately consider the synthesis variables to precisely adjust the morphology and structure of the oxide for the desired material properties in a specific application.

According to this, a relatively important parameter affecting the final morphology of anodic nanostructures described in the literature is the anodization mode, either potentiostatic or galvanostatic.^9^ Even though anodization under a potentiostatic regime is still the most widely used method for creating nanostructures, galvanostatic oxidation possesses its own benefits. For one, it allows for the formation of porous morphologies at low current densities, making it a more encouraging option when considering scaling up the process.^10^ The other advantage of the former lies in the possibility of controlling the charge flowing through the electrode, which leads to an unchanging reaction rate during the initial stages of oxidation and as a result improved control of the growth of the fabricated nanostructure. According to the literature, altering the anodization mode to galvanostatic also affects an oxide growth rate^10,11^ and alleviates partial shaping of the resultant nanostructure arrangement.^12^ For instance, studies concerned with Zn anodization revealed that galvanostatic oxidation significantly increases the amount of nucleation sites formed at the initial stages of the oxidation process.^13^ The growth and architecture of anodic tin oxide through galvanostatic anodization have been studied; however, resultant work concerns anodization in acidic electrolytes.^14^ Moreover, information about its morphological evolution and photoactivity still remains limited.

The electrochemical polishing procedure is often employed as a pretreatment step of anodization also known for impacting the architecture of the final nanostructure. Indeed, besides removing the native oxide layer from a metal surface, the polishing offers other important benefits, such as improvement of nanostructure organization, as proven in numerous works concerned with anodic TiO_2_ or Al_2_O_3._^15,16^ Even though the polishing procedure of Sn has been utilized before,^17^ the current literature does not provide research regarding its impact on the arrangement, morphology, and properties of the anodically grown oxide layers.

From this background, in the current study, we investigated the growth and morphology of anodic tin oxide obtained via galvanostatic anodization conducted for various process durations using diverse foil purities and by partially implying an electrochemical polishing procedure. Additionally, the influence of Sn substrate purity, electropolishing pretreatment procedure, and annealing on the optical and photoelectrochemical properties of the synthesized materials was elucidated and discussed.

## Experimental Section

Tin foil specimens (98.8 and 99.9% purities, Goodfellow) with dimensions 1 cm × 2 cm were degreased and cleaned in acetone and ethanol and then partially subjected to the electrochemical polishing procedure in an acetic acid–based electrolyte containing HClO_4_ (16.8 vol %) and water (3.4 vol %) for 120 s at 0 °C. After that, the working surface area was defined using an acid-resistant paint. In the next step, nanoporous SnO_*x*_ layers were fabricated via a one-step anodic oxidation process carried out under a galvanostatic regime with a current density of 20 mA·cm^–2^ for diverse times (5, 10, 30, 60, and 90 min). In our previous works, we demonstrated that anodization carried out at 4 V in 1 M NaOH enabled obtaining crack-free nanoporous SnO_*x*_ layers; therefore, in this research, we conducted the galvanostatic oxidation at the given current density, which corresponds to the steady-state current observed at this potential. Afterward, the samples were partially subjected to thermal annealing in air at 200 °C for 4 h with a heating rate of 2 °C·min^–1^ using a muffle furnace (FCF 5SHM Z, Czylok).

The morphology of obtained nanostructured layers was examined using a field emission scanning electron microscope (FE-SEM/EDS, Hitachi S-4700 with a Noran System 7), and the features of nanostructures (i.e., pore diameter and thickness) were estimated directly from FE-SEM images using the scanning probe image processor WSxM v.12.0.^18^

Atomic force microscope (AFM) topography images were obtained with Dimension Icon XR microscope (Bruker, Santa Barbara, CA) working in the air in the PeakForce Tapping (PFT) mode, using standard silicon cantilevers with a nominal spring constant of 0.4 N/m, triangular geometry tip, and a nominal tip radius of 2 nm. For AFM working in the water, a silicon cantilever with a normal spring of 0.7 N/m, triangular geometry tip, and a nominal tip radius of 2 nm was used.

UV–vis diffuse reflectance spectra (DRS) were measured using a Lambda 750S spectrophotometer (PerkinElmer, Waltham, MA) equipped with an integrating sphere module (in the range of 250–800 nm with a step of 2 nm) using Spectralon SRS-99-010 as the reference.

Photoelectrochemical experiments were carried out in a three-electrode cell with a quartz window with Sn/SnO_*x*_ samples, Pt foil, and saturated calomel electrode (SCE) as the working, counter, and reference electrodes, respectively, in a borate buffer (pH ∼7.5) electrolyte at the applied potential of 1 V vs SCE under chopped light irradiation (30 s on/30 s off per cycle) for 50 cycles. As a light source, a solar irradiation simulator (Insytut Fotonowy, Krakow, Poland) equipped with a 150 W xenon arc lamp was used.

## Results and Discussion

**Figure 1:**
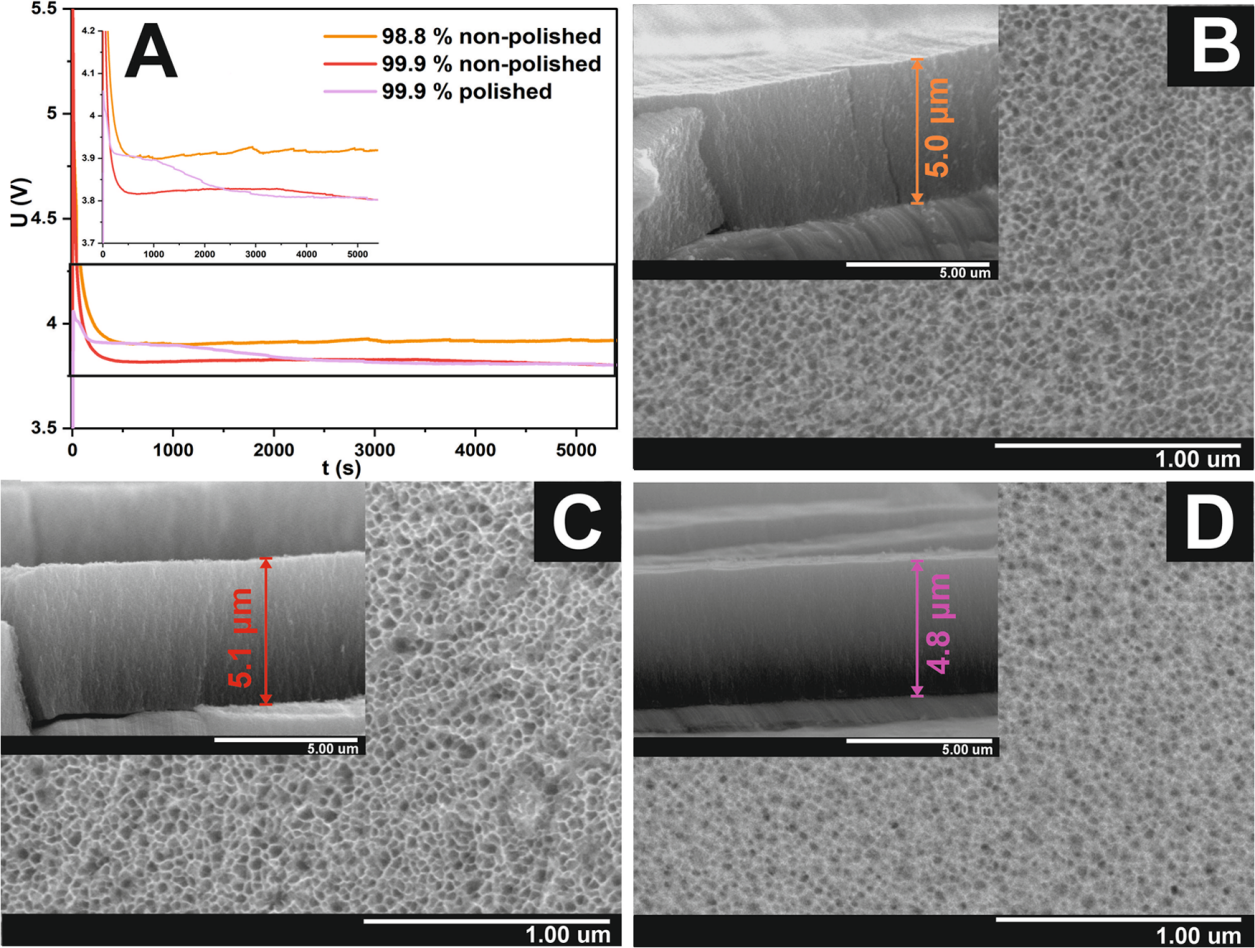
Potential vs time curves recorded during anodic oxidation of 98.8% Sn (orange line), 99.9% Sn nonpolished (red line), and 99.9% Sn polished (purple line) foils in 1 M NaOH recorded during 90 min anodization (A). FE-SEM images of surfaces and cross sections (insets) of anodic tin oxides fabricated on 98.8% (B), 99.9% nonpolished (C), and 99.9% polished (D) substrate during the processes lasting for 60 min. In all cases, anodization was carried out at a current of 20 mA·cm^–2^.

First, the influence of the electrochemical polishing procedure on the surface of the Sn foil was verified using atomic force microscopy (see Figure S1a and b in the Supporting Information). Based on the micrographs, the beneficial impact of the polishing can be visible: the metal surface is free from protrusions or traces after rolling and smoothened as evidenced by calculated surface roughness (*R*_q_) of 2.5 ± 0.6 and 15.9 ± 2.9 nm for the polished and unpolished surfaces, respectively. Figure 1A presents voltage vs time curves obtained during the anodic oxidation conducted on diverse metallic Sn substrates in 1 M NaOH electrolyte for 90 min at the current density of 20 mA·cm^–2^. First, in all cases, the recorded voltages are slightly below the value of 4 V, which is in line with the results observed during potentiostatic anodization including our previous works.^19^ Moreover, anodization of Sn foil with higher purity led to marginally lower voltage in comparison to a 98.8% Sn substrate, which can suggest that the oxide layer growth on a higher-purity surface is more efficient, as previously observed in the case of other anodic oxides.^20^ The courses of curves recorded during the anodization of nonpolished Sn foils indicate that oxide growth proceeds comparably with those obtained under potentiostatic mode.^19^ After the initial formation of the compact oxide film, a generation of nanoporous oxide gradually occurs during the first 500 s of the anodic oxidation, resulting in the decay of the recorded potential. After this time, the potential value remained almost constant during the whole process. On the other hand, regarding the curve courses, anodization carried out on the electropolished Sn surface proceeds differently: the observed voltage decreases gradually for more than 2000 s from the beginning of the process. A possible reason for this phenomenon could be the absence of grain domains and discontinuities on the polished surface. Since these irregularities may be perceived as areas where the oxide layer is more likely to crack under stress, it is evident that with a more uniform and compact oxide layer (as found on the polished surface), the material is able to withstand the stress more effectively.

**Figure 2:**
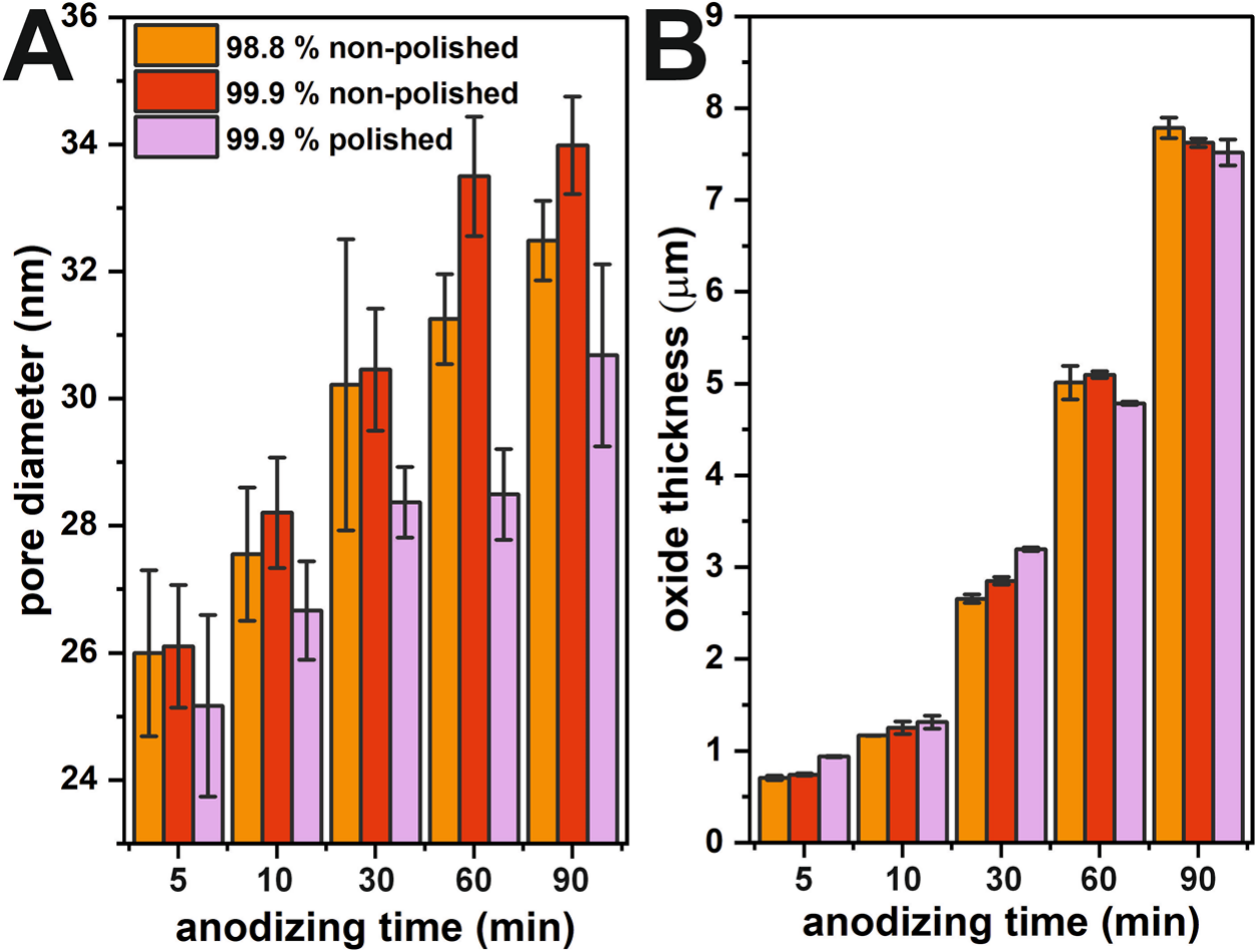
Averaged pore diameters of the anodic oxide layers fabricated on diverse substrates (A) and thicknesses of the anodic films estimated from the cross-sectional images (B) as a function of oxidation time.

FE–SEM images of the surfaces of nanoporous SnO_*x*_ layers fabricated during 60 min of anodization of different metallic Sn substrates in 1 M NaOH are presented in Figure 1 B and D. As can be seen, a nanoporous form of the oxide is present in all studied conditions, independently of the substrate used. Since the regularity of the formation of the pores is independent of Sn substrate type, it is evident that implementation of the electropolishing procedure does not trigger more organized pore distribution unlike in cases of Al_2_O_3_ or TiO_2_, which is consistent with other works concerning anodic SnO_x_.^17^ Nonetheless, the polishing pretreatment alleviated the fabrication of a more continuous layer with no cracks resulting from the presence of grain boundaries unlike layers grown on unpolished surfaces (see Figures S1A, B and S2 in the Supporting Information). The cross-sectional view images (insets) of fabricated materials also indicate that nanoporous layers are continuous and free of transverse cracks resulting from oxygen release during oxidation as also presented in the literature.^19^*R*_q_ values estimated for tin oxide layers obtained in a 30 min process on a polished and unpolished Sn foil were 5.1 ± 0.7 and 11.2 ± 2.4 nm, respectively. This proves that layers obtained on a smoothed substrate are also less rough. Subsequently, the morphological features (pore diameter and oxide thickness) for materials fabricated on diverse Sn substrates depending on the anodizing time (5–90 min) estimated from the micrographs are collected in Figure 2A,B, respectively.

Primarily, in Figure 2A, a pore diameter widening is observed with increasing the anodization duration. The collected data show that the type of foil used affects the size of the pores, and the smallest ones were observed in the nanoporous SnO_*x*_ grown on the polished foil. Moreover, the longer the anodizing duration, the greater effect of electropolishing on the pore size is observed. This finding confirms that the electropolishing pretreatment helps in achieving more uniformly arranged and densely packed nanochannels.

**Figure 3:**
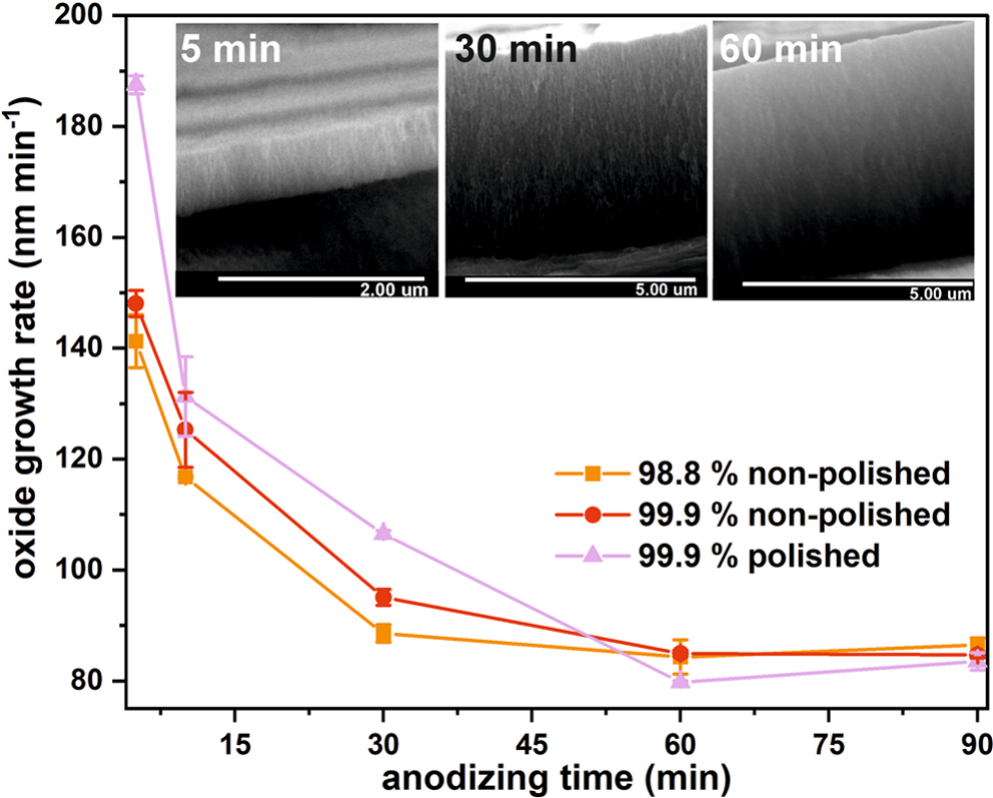
Oxide growth rate calculated as a function of anodization time for all studied materials with an example cross-sectional images of 98.8% SnO_*x*_ anodized for 30, 60, and 90 min, respectively (inset).

As expected, increasing the time of anodization results in obtaining an anodic layer with a greater thickness independent of the substrate type. In the case of electropolished foil, the oxide growth process seems to be accelerated when compared with nonpolished 98.8 and 99.9% Sn regarding anodizations lasting 5, 10, and 30 min. Since the electropolishing procedure results in a smoothed surface and removes debris, residues, and the passive oxide layer on the metallic substrate, the oxide layer formation is facilitated. The values of the oxide growth rate were fairly in line with those observed during the initial 10 min of anodization carried out at the constant potential of 4 V (at which, as explained above, similar current densities were recorded) under the same anodizing conditions (electrolyte composition, temperature, etc.). Based on the estimated oxide growth rates (Figure 3), it is seen that when the anodization proceeds, the oxide layer growth becomes more suppressed. Independently on the Sn foil, when the process is further elongated up to 120 min, semiconducting film growth is even less effective (estimated thicknesses of the oxides were in a range of 8.4–8.6 μm, depending on the substrate type, for sample morphology, see Figure S2 in the Supporting Information). Considering that the anodization is carried out at a constant current density, the lower rates of oxide formation observed for longer anodizing duration could potentially suggest that after a certain time, the greater charge is consumed for other (than oxide film growth) reactions, like oxygen evolution, especially since this process is the main factor responsible for the formation of nanochannels within the anodic tin oxide films.^19,20^ However, as can be seen in Figure 1A, no increase in electrode polarization is observed, which suggests that diffusion limitations concerned with hindered access of the electrolyte to the metallic substrate are not the main reason for the observed phenomena. Therefore, a gradual thinning of the oxide layer caused by chemical etching in strongly alkaline electrolytes must be the main factor responsible for the oxide growth rate decrease.^21^ A similar phenomenon was observed in our group for the potentiostatic anodization of Sn.^7^ Finally, considering that the higher values of anodizing potential were recorded during the initial stages of anodization, the values of electronic and ionic currents responsible for O_2_ evolution and oxide formation may change even if the total current remains unchanged. However, since for potentiostatic anodization it is possible to distinguish both currents from current density vs time curves,^22−25^ in the case of the galvanostatic process, it is not a trivial issue and requires some further investigations.

**Figure 4:**
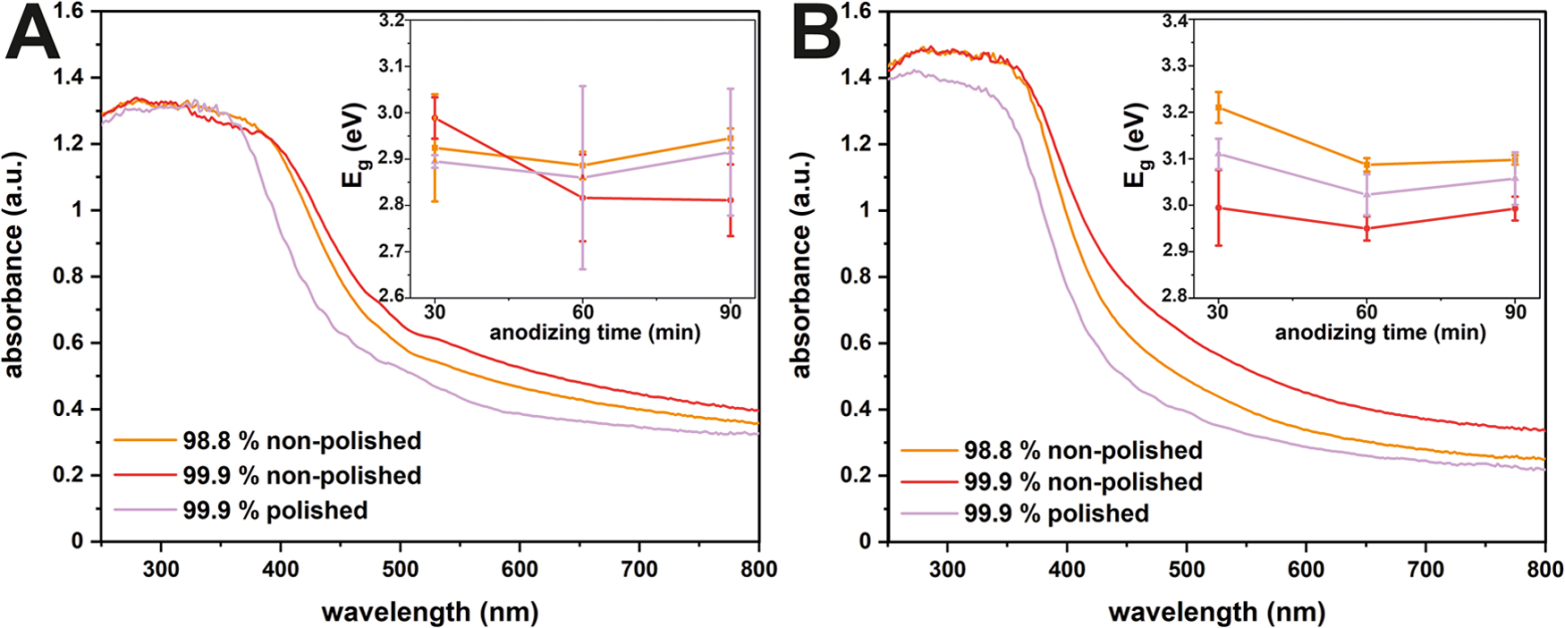
Absorbance spectra calculated from the DRS data of amorphous (A) and annealed (B) anodic tin oxide layers fabricated on all studied substrates for 30, 60, and 90 min together with their estimated optical band gaps (insets).

After that, the optical properties of the studied materials were verified by DRS measurements before and after applying the thermal treatment procedure. First of all, materials obtained during the 5 and 10 min anodizations were not included in the analysis due to the occurrence of Fabry–Pérot interferences caused by Sn interaction with light.^26^ These interferences significantly affect the accuracy of the results and, therefore, were excluded from further analysis (e.g., absorbance spectra, see Figure S4 in the Supporting Information). Example spectra recorded for materials grown on diverse substrates for 60 min before and after annealing are shown in Figure 4. It is seen that regardless of the substrate type, all fabricated nanoporous layers exhibit a significant absorption in the UV region with an exponential course near the optical band edge. Even though the absorption edge occurs within a lower wavelength in the case of SnO_*x*_ grown on a polished surface, the shape of the curves can be assigned to poorly crystalline anodic tin oxide.^8^ As can be observed, over the entire wavelength range, all materials show a nonzero absorption value, which is probably due to the amorphous and highly defective nature of the nanoporous oxide. After the annealing procedure was applied, the absorbance values increased within the ultraviolet range, and the absorption edge underwent a hypsochromic shift independently of substrate purity and the electropolishing procedure. This may suggest that thermal treatment contributed to an increase in the stoichiometric form of SnO_2_; however, as already proven in numerous works, an amorphous structure still remains predominant after annealing at 200 °C.^7,27^

To gather a more quantitative insight into this phenomenon, the optical band gaps of the synthesized samples were calculated (insets of Figure 4) using the following Tauc equation:
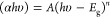
where α is the molar extinction coefficient, *hν* is the energy of light (eV), *A* is the material parameter, *E*_g_ is the band gap of the sample (eV), and *n* is a constant dependent on the type of transition.

The obtained results display a distinct correlation with the recorded shifts of the absorption edge presented in Figure 4. Specifically, the band gap values decrease as the anodic oxidation time increases, whereas electropolishing and thermal treatment contribute to notable increment in the *E*_g_.

**Figure 5:**
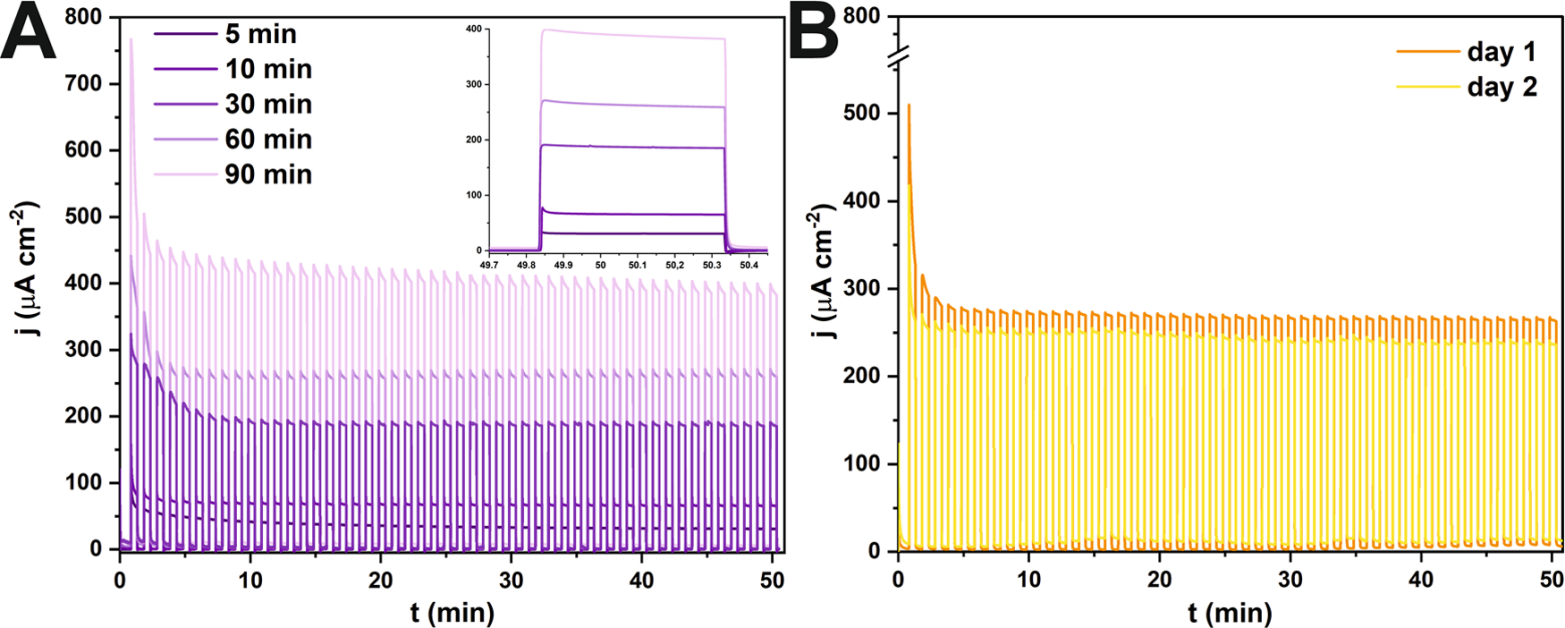
Chronoamperometric curves recorded under chopped irradiation with a 1.5 G solar simulator (30 s on/30 s off) for SnO_*x*_ nanoporous layers grown on electropolished Sn subjected to annealing procedure (A) and chronoamperometric measurements performed for nonannealed anodic SnO_*x*_ obtained via 60 min anodization of polished 99.9% Sn over 2 days (B).

Subsequently, photoactivity of all fabricated tin oxide layers was investigated using a 1.5 G solar light simulator, and exemplary chronoamperometric curves recorded for SnO_*x*_ layers grown on the polished surface for diverse times are presented in Figure 5A (the results of all of the measurements are collected in Figure S5 in the Supporting Information). As observed, the photoresponse of the studied materials increases with the thickening of the anodic film layer which is in line with the results obtained for potentiostatically fabricated SnO_*x*_ nanoporous layers.^7^ In the inset of Figure 5A (extracted 50th illumination cycle), it is seen that with increasing thickness of the nanoporous layer, the rise time of the photocurrent also elevates. Regarding literature, this shape might be attributed to the fact that the thicker layers exhibit enhanced recombination within the space charge layer, which may affect resultant electron transport.^28,29^ Interestingly, independently of the Sn substrate type and anodizing time, a district photocurrent transient decay that persists over the first few irradiation cycles can be distinguished.

To verify the stability and reversibility during the PEC performance, the photoactivity of studied materials was tested over time and exemplary chronoamperometric curves recorded on two different days are collected in Figure 5B. Upon retesting the same sample for the measurement, it was observed that the material exhibited similar photoactivity. Comparing both curves, it can be stated that the photoresponse of the sample is stable as indicated by only ca. 10% decay of the steady-state photocurrents and after a total of 2 h of measurement. The initial photoactivity drop may originate from recombination taking place within the structure under illumination until equilibrium is reached. On the other hand, considering results recorded for the annealed materials, it is evident that the time needed to attain the equilibrium is even elongated, which indicates that the photoelectrochemical behavior can be additionally affected by applying this post-treatment procedure.

**Figure 6:**
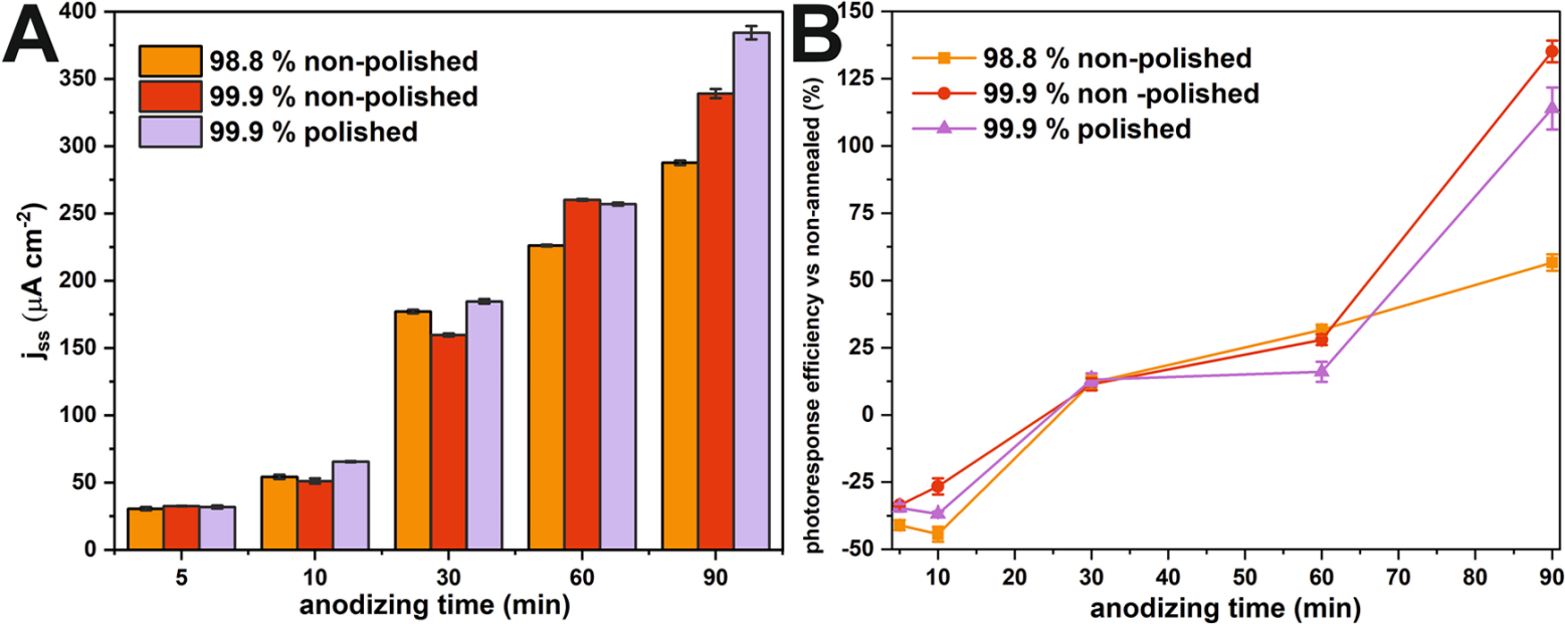
Steady-state photocurrents subtracted from the chronoamperometric curves collected for the studied materials after the thermal treatment (A) and efficiencies of annealing as a function of anodization time (B).

Afterward, steady-state photocurrent values of the annealed samples were extracted from the chronoamperometric curves (Figure 6A). It can be observed that the substrate purity and electropolishing do not significantly affect the photoactivity of materials grown during 5 and 10 min anodizations; however, the substrate type becomes non-negligible with increasing anodization time. Given that the primary impurity in the tested Sn foils is antimony (see the Supporting Information), it is reasonable to assert that its presence could be the factor influencing photoelectrochemical (PEC) properties.^30^ Nonetheless, analyses of the composition of anodic tin oxide present in the literature^31,32^ have not evidenced the presence of structural components beyond tin and oxygen (or phosphorus if the process is conducted in orthophosphoric acid). This suggests that utilizing a lower purity substrate does not result in structural modifications substantial enough to significantly impact the PEC properties.

Among the studied conditions, the SnO_*x*_ layers grown on the electropolished surface exhibit the highest photocurrents among all of the studied samples. Considering the positive influence of an electrochemical polishing procedure, it is worth highlighting that this pretreatment procedure partially removes and smooths the grain boundaries of the metallic Sn. According to the literature,^33^ on both sides of grain boundaries, the formation of a potential barrier is triggered. This phenomenon accelerates recombination between photoinduced electrons and holes after reaching a grain boundary charge layer. Hence, the obtained results indicate that the fabrication of SnO_*x*_ layers on an electropolished surface partially defeats issues concerned with charge carrier recombination increasing its overall PEC performance.

Referring to the literature on other anodic oxides,^34,35^ it seems that substrate purity significantly impacts the photoelectrochemical properties. Regarding anodic SnO_*x*_, a similar trend can be observed, especially for the thicker layers. The reversed trend observed for 5, 10, and 30 min anodizations may originate from additional contributions of impurity elements, which elevate the photocurrents in the case of thin layers, but start to limit the photoactivity by impairing charge transfer in thicker SnO_*x*_ layers.

Afterward, the influence of thermal post-treatment on the photoactivity of studied materials was verified (Figure 6B) and depicted as an efficiency of photoresponse of the annealed samples vs nonannealed after reaching a steady-state value. After conducting a thorough analysis, it was observed that when the thickness of the layers is less than 2 μm, there is a significant decrease in photoactivity after annealing. However, when the layers are thicker, there is an improvement in performance after undergoing the post-treatment process. Despite the overall improvement in efficiency in photoactivity, the lowest increase in efficiency was recorded for SnO_*x*_ layers grown on 98.8% Sn purity, which is in agreement with the results already present in the literature^34^).

The photocurrents generated by the SnO_*x*_ photoanode on the polished 99.9% Sn substrate exhibit a significant increase when compared with those observed for the photoelectrode on the unpolished and low-purity foil. On the other hand, the cost-effectiveness of utilizing higher-purity foils and electropolishing is uncertain when assessing both the obtained values and the prices of individual anodization substrates. Thus, it is crucial to meticulously consider both factors when designing the anodizing procedure to achieve nanoporous photoelectrodes with an optimal photoelectrochemical performance.

## Conclusions

In this study, for the first time, the effect of the Sn foil purity used for the anodization of nanoporous tin oxide was examined in detail for anodizing times in a range between 5 and 90 min. The studies revealed that foil purity and electropolishing possess a significant impact on morphological features (pore diameter and oxide film thickness); however, in all cases, the material exhibited randomly distributed pores. The results of our study suggest that the characteristics of the Sn substrate have a notable influence on the optical and photoelectrochemical properties of the obtained oxide films. Specifically, the type of Sn substrate used plays a significant role in determining the resultant outcome of these properties. The findings of this research indicate that careful consideration of the Sn substrate choice is crucial for obtaining the desired results in related fields.

## Supporting Information Available

AFM images of polished and unpolished Sn and SnO_*x*_ surface; SEM images of polished and unpolished Sn; surface and cross-sectional images of anodic SnO_*x*_ layers grown during a process lasting for 120 min; absorbance spectra of amorphous anodic SnO_*x*_ obtained during anodization lasting for 5 and 10 min; chronoamperometric curves recorded for all of the studied materials during sequential illumination of solar simulator 1.5 G lamp (PDF)
