# Supplementary material for: Effect of Sn Purity and Electropolishing Procedure on the Morphology and Properties of Nanoporous SnOx Layers Obtained via Galvanostatic Anodization
Source: ACS Omega. 2025 Jan 14;10(3):2442–9. doi: 10.1021/acsomega.4c03630 (PMC11780425; doi:10.1021/acsomega.4c03630)
Supplement: Supplementary file 1 — ao4c03630_si_001.pdf [file ao4c03630_si_001.pdf]

Supplementary Information

Effect of Sn purity and electropolishing procedure on the morphology and properties of nanoporous SnO_x layers obtained via galvanostatic anodization

Magdalena Gurgul*, Bernadetta Macuda, Tomasz Kuciel, Leszek Zaraska

Jagiellonian University, Faculty of Chemistry, Gronostajowa 2, 30-387 Krakow, Poland

Keywords: tin oxide; nanoporous layers; anodic oxidation; photoelectrochemical activity

* Corresponding author. E-mail: gurgulm@chemia.uj.edu.pl (M. Gurgul)

Faculty of Chemistry, Department of Physical Chemistry & Electrochemistry,

Jagiellonian University

Gronostajowa 2, 30387 Krakow, Poland

Composition and percentage content of Goodfellow Sn foil 98.8 % purity:

Sb (1.1 %), Cd (0.001 %), As (0.002 %), Cu (0.01 %), Fe (0.001 %), Ni (0.001 %), Zn (0.001 %), Ag (0.001 %), Pb (0.06 %), Bi (0.01 %).

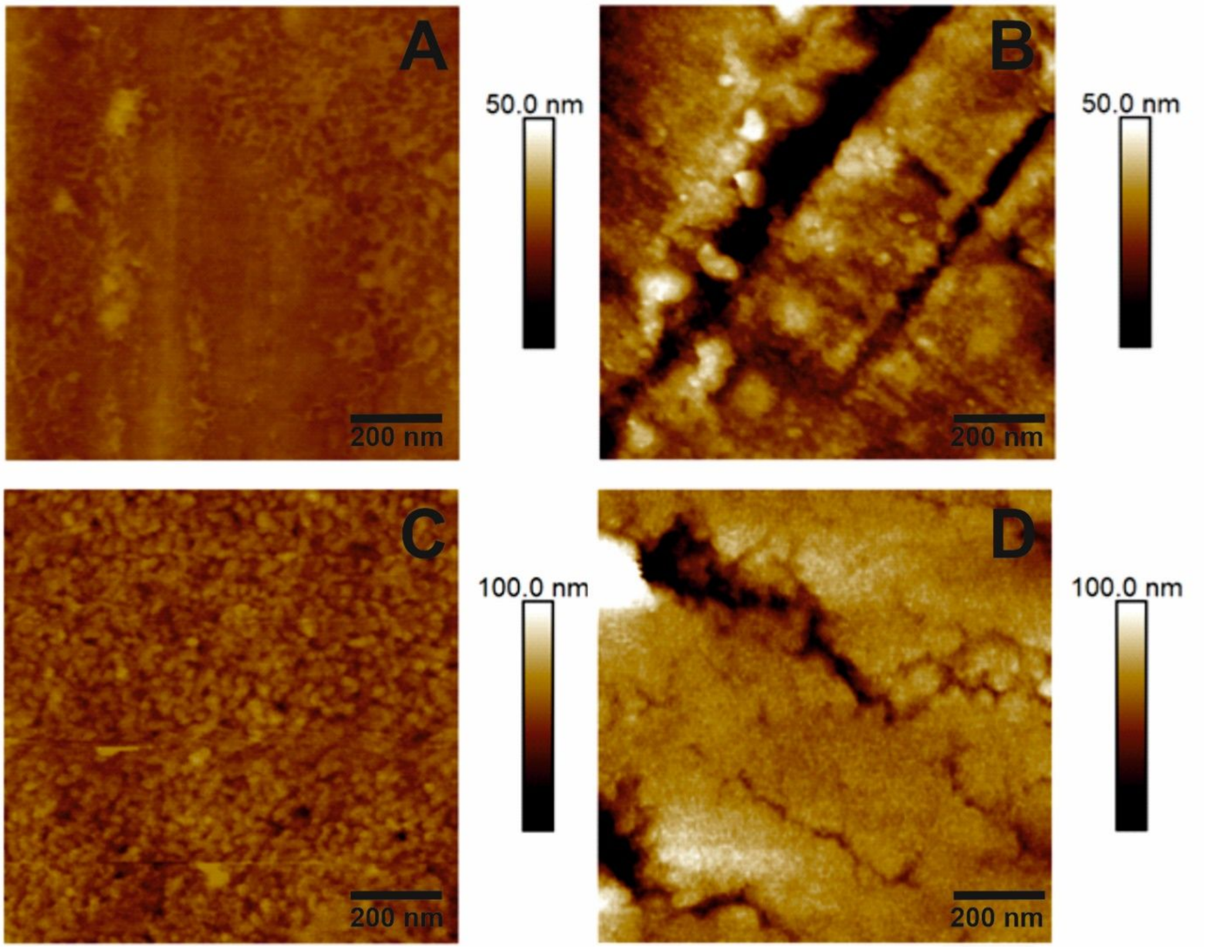

Figure S1 AFM images of Sn surface before (A) and after (B) electrochemical polishing procedure and SnO_x grown during the process carried out for 30 min on polished (C) and unpolished (D) surface.

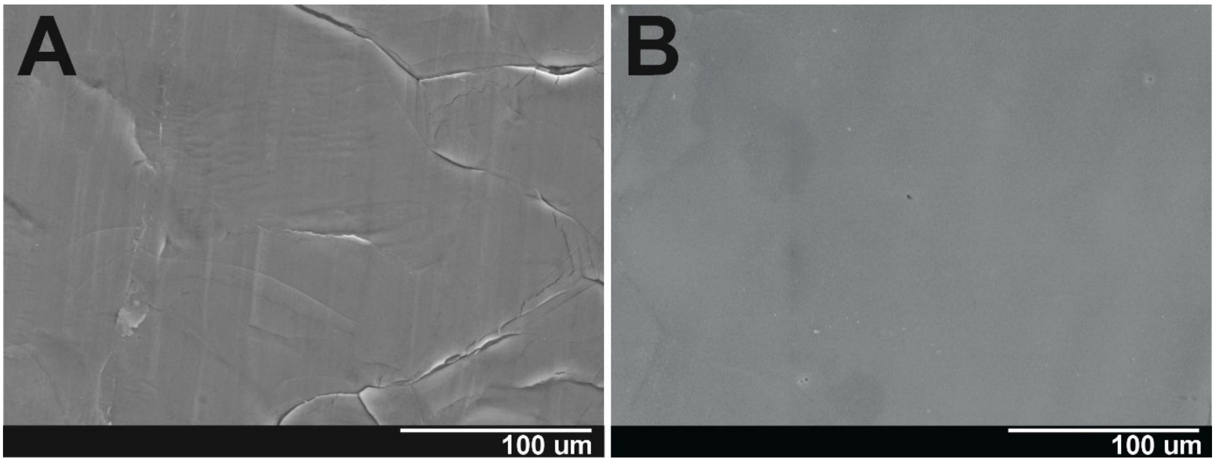

Figure S2. Low-magnification FE-SEM images of anodic tin oxide layers fabricated via galvanostatic anodization of non-polished (A) and polished (B) 99.9 % purity foil for 90 min.

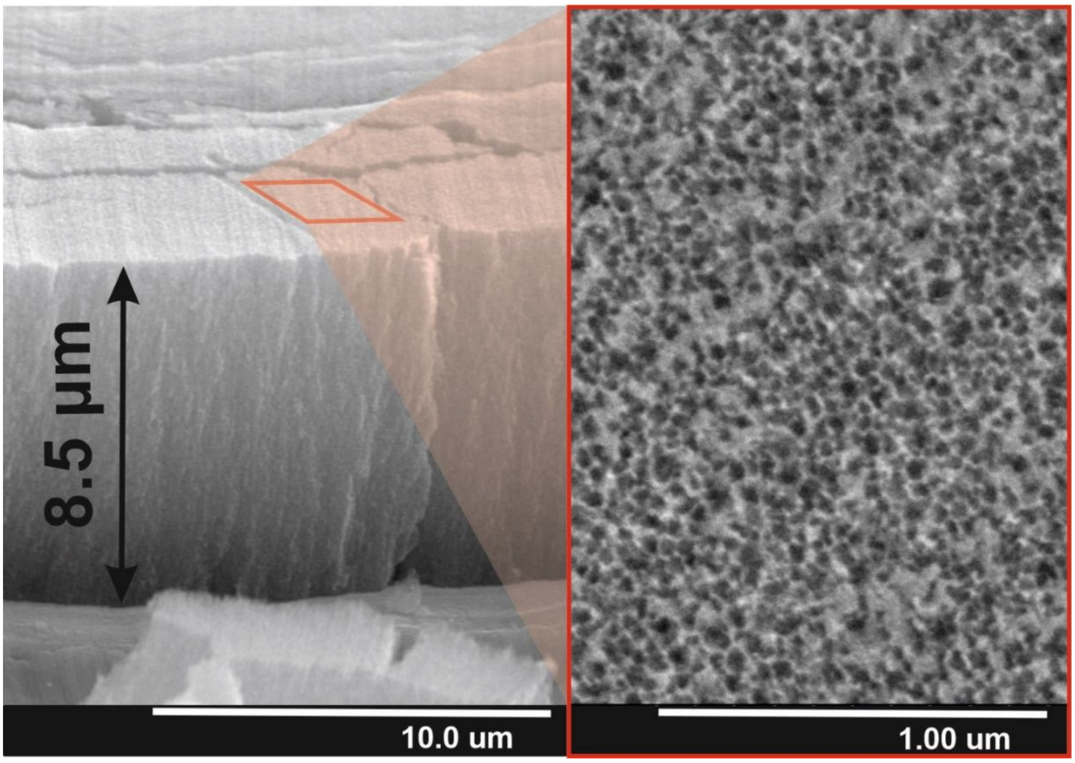

Figure S3. FE-SEM surface and cross-sectional images of tin oxide layer anodically grown on the 98.8 % Sn foil for 120 min in an alkaline electrolyte.

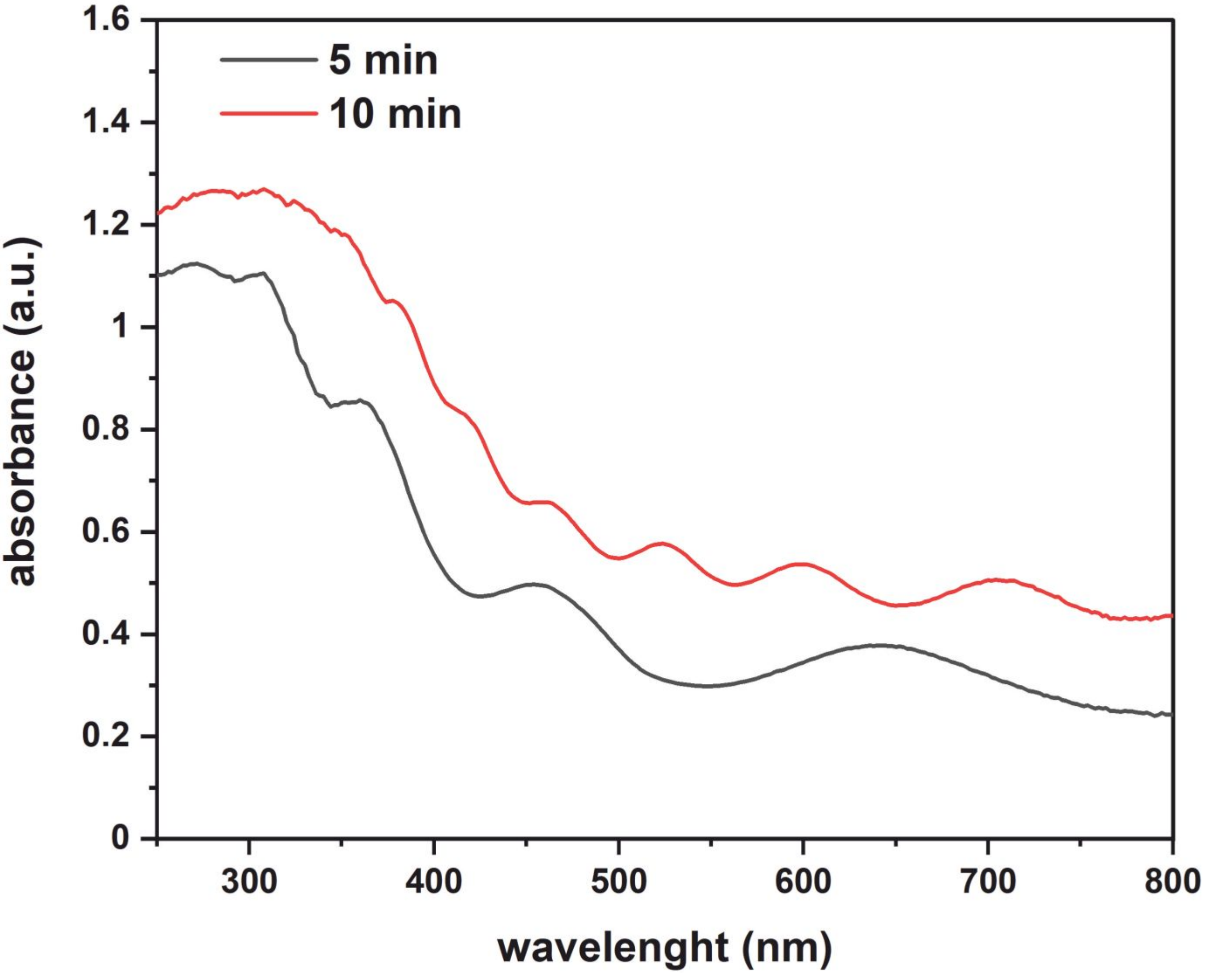

Figure S4. Absorbance spectra of amorphous anodic tin oxide layers fabricated on 98.8 % substrate for 5 and 10 minutes.

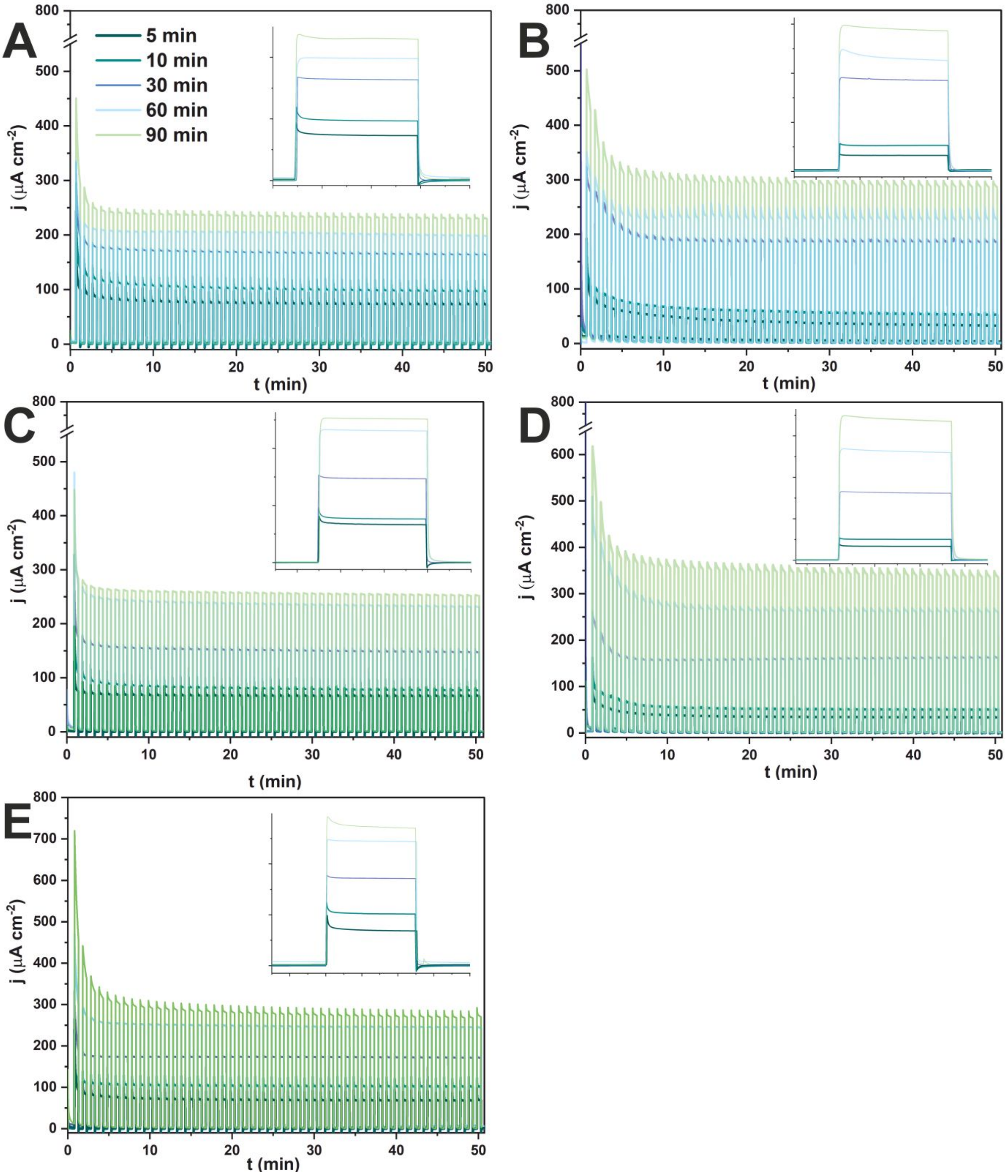

Figure S5. Chronoamperometric curves collected for SnOx layers grown on 98.8 % Sn foil (A, B), 99.9 % non-polished foil (C, D), and 99.9 % polished Sn foil (E) before (A, C, D), and after the thermal post-treatment (B, D).
